# Real-Time Condition Monitoring System for Electrode Alignment in Resistance Welding Electrodes

**DOI:** 10.3390/s22218412

**Published:** 2022-11-01

**Authors:** Daniel Ibáñez, Eduardo García, Jesús Soret, Julio Martos

**Affiliations:** 1 Department of Electronic Engineering, Campus de Burjassot, Universidad de Valencia, CP 46100 Valencia, Spain; 2 Ford Spain, Poligono Industrial Ford S/N, Almussafes, CP 46440 Valencia, Spain

**Keywords:** resistance spot welding, magnetic field, condition monitoring, welding quality

## Abstract

Electrode misalignment, produced by mechanical fatigue or bad adjustments of the welding gun, leads to an increase in expulsions, deformations and quality problems of the welding joints. Different studies have focused on evaluations of the influence of a misalignment of the electrodes and the final quality of the weld nugget. However, few studies have focused on determining a misalignment of the electrodes to avoid problems caused by this defect, especially in industrial environments. In this paper, a method for performing the condition monitoring of electrode alignment degradation was developed following previous research, which has shown the relationship between the misalignment of short-circuited electrodes and the magnetic field generated by them. This method was carried out by means of a device capable of measuring the magnetic field. Finally, an integral system for the detection of misalignments in real production lines is presented. This system set behavior thresholds based on the experimentation, allowing the condition monitoring of the alignment after each welding cycle.

## 1. Introduction

The welding process is one of the most used metal joining methods in the manufacturing industry. Specifically, in the automobile manufacturing process, it represents more than 90% of all the welded joints of the bodywork [1,2,3]. The resistance welding process bases its operation on Joule’s law, which states that when a current flows through the metal to be melted with a certain resistance, heat is generated that melts the metal, causing the welded joint [4,5,6].

**Figure 1:**
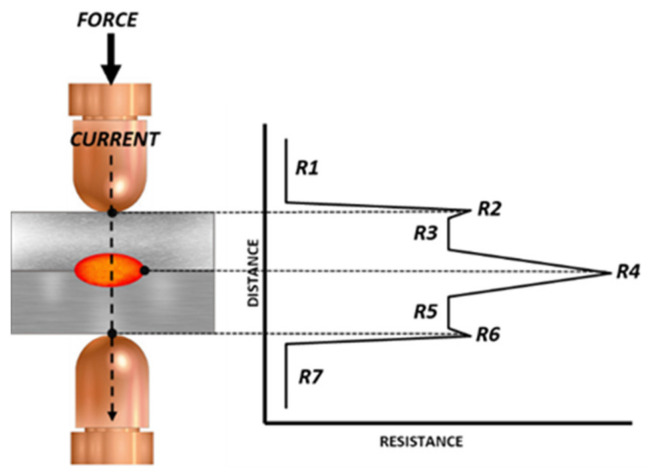
Layout of the resistance welding process.

Figure 1 shows the standard layout of a resistance welding spot, in which two copper electrodes apply pressure and a current flows through the metal sheets to be welded. In the manufacture of a car body, the metals to be welded are usually steel, with a resistance greater than the resistance of the copper electrodes. As a result, heat is primarily generated in the metals to be welded. In addition, as can be seen in Figure 1, the contact resistance between the sheets, R4, is even greater than the resistance of the sheets themselves, R3–R5. This allows a nugget to begin to be formed between the metal sheets. The electrical circuit can be summarized in seven resistances from the flow of the current from one electrode to another: the resistance of the electrodes (R1 and R7); the contact resistance of the electrode and the metal sheet (R2 and R6); the self-resistance of the metal (R3 and R5); and the contact resistance of the metal sheets [7,8,9].

Bearing in mind these seven resistances, it can be affirmed that each one of these resistances has a fundamental role in the generation of heat and its distribution during the welding process. In short, each one of these resistances has a crucial role in the weld quality.

Consequently, different authors have investigated how to guarantee or evaluate the quality of a weld based on these resistances, mainly in those resistances that are more critical and variable such as the contact resistance between the electrode and the sheet (R2 and R6) and the resistance caused by contact between the sheets (R4). Regarding contact resistance, Chen et al. [10] showed a coating designed as a barrier to prevent the electrodes from alloying with the Zn coating and causing degradation by pitting or erosion, reducing the variations in the contact resistance. Other studies have shown the importance of keeping the geometry of the electrodes constant to reduce variations in the diameter of the active face of the electrodes, either due to excess dirt or mushrooming [11,12,13,14,15,16]. In the same way, the contact resistance between metals (R4) has a great importance in the different research that has been carried out; i.e., different authors have focused their studies on the influence that the gap between sheets has on the final quality of the welding point [17,18,19].

In this study, we focused on the detection of problems in the contact resistance of electrodes, specifically those related to the alignment of the electrodes.

### Electrode Misalignment

**Figure 2:**
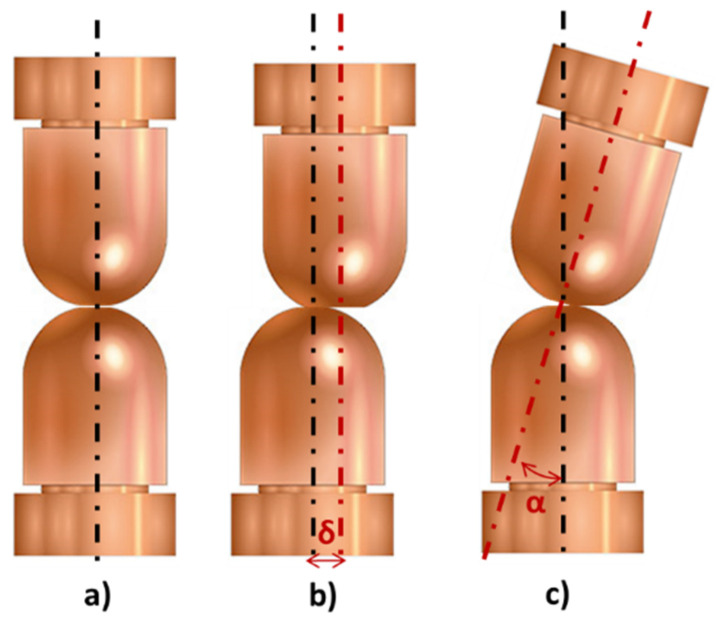
Electrode alignment status. (**a**) Aligned electrode. (**b**) Axial misalignment. (**c**) Angular misalignment.

Eventually, due to mechanical fatigue, electrodes lose their working axis, presenting angular (α) or axial (δ) displacements with respect to their reference axis. This defect, shown in Figure 2, is known as misalignment. An electrode misalignment provokes unfavorable characteristics during the welding process as well as in the quality of the welded joints. Misalignments, whether axial or angular, can cause irregularly shaped welds as well as a reduced weld size and projections due to an asymmetric distribution of the force and a change in the contact surface [20,21,22,23,24,25,26,27,28].

This research focused on the detection of an axial misalignment, as shown in Figure 2b, because it is more common in production lines but it could be extrapolated to any type of misalignment. As mentioned previously, a modification of the contact surface is caused by a misalignment and it is expressed by the equation of the contact surface (Ca) (Equation (1)), from which it can be deduced that the greater the misalignment, the smaller the contact surface between both electrodes.

(1) $$C_{a}=2r^{2}\sin^{-1}\left(\sqrt{1-{\left(\frac{\delta }{2r}\right)}^{2}}\right)-\delta \sqrt{r^{2}-{\left(\frac{\delta }{2}\right)}^{2}}$$

where *r* represents the radius of the tip of the electrodes and δ is the misalignment between the electrodes.

Hence, the alignment of welding electrodes has a major impact on the quality of the spot welding joint. As a result, it is fundamental to analyze the alignment condition for each welding gun.

## 2. Research Objectives

The aim of this research was not only to find a relationship between misalignments and a measurable physical variable, but also to find a complete system for measuring and detecting the alignment state in real-time that was applicable to high-production factories by means of resistance welding.

Several authors have proposed methods for the detection of electrode misalignments in the resistance welding process. Li et al. [29] discovered a method for detection by means of an image analysis. The results obtained by this method suggested that it was possible to determine if the electrodes were properly aligned. The main disadvantage of the use of images resides in the cost of the implantation of the cameras and their integration into productive lines. In addition, due to the environment in which the cameras are located, continuous maintenance is needed to prevent the lenses from getting dirty or the measurements from being miscalibrated. For these reasons, the implementation of this method in a high-production factory is not feasible. On the other hand, Xing et al. [30] presented in their research different methods to determine the state of alignment of the electrodes. One was based on the analysis of images of the carbon footprint of the electrodes, presenting the same disadvantages as the previous method; a second method studied the displacement of the electrodes and the applied force. The main drawback of this last method was that not only was the misalignment of the electrodes analyzed, but also the general conditions of the state of the electrodes, making difficult to determine if the change in conditions was due to a misalignment or other mechanical defects. Finally, Lee et al. [31] proposed a method for the detection of an angular misalignment by means of machine learning. Specifically, the proposed method used the signals of dynamic resistance, voltage and current to extract the critical features for training a neural network. Despite the good results of this research, the high processing and computing capacity required for each welding gun makes its installation complex in a high-production industry, necessitating training for each welding gun.

Therefore, the objective of this research was to find a method for the detection of misalignments. This method had to be robust, implantable and focused on the alignment of the electrodes.

**Figure 3:**
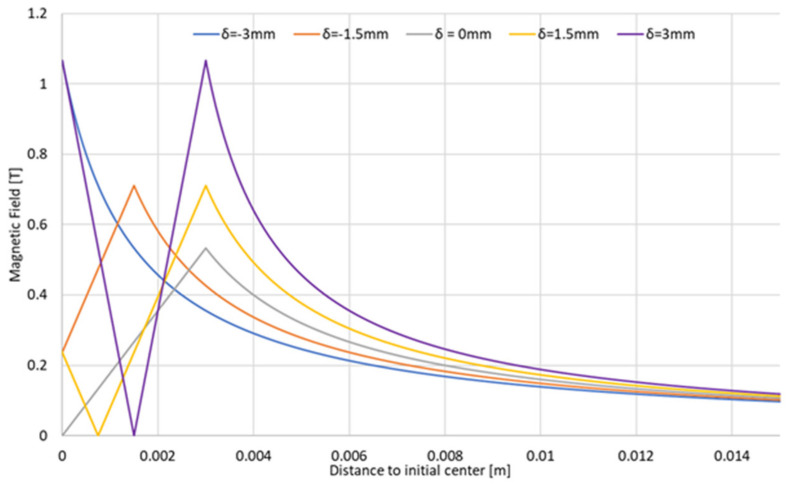
Magnetic field generated as a function of the state of alignment of the electrodes.

In previous investigations, an initial method for the detection of misalignments by measuring magnetic fields was proposed. First, the theoretical influence between the state of the alignment of the electrodes and the magnetic field generated by them was investigated [32]. By reducing the electrodes to a wire with a constant current density, it could be stated that the behavior of the generated magnetic field followed Ampere’s law [33,34], where the magnitude of the magnetic field fundamentally depends on the distance toward the origin of the magnetic field; in this case, the center of the contact surface of the electrodes. When a misalignment appeared, the center of the magnetic field moved in the same direction as the misalignment. This provoked an asymmetry in the generated magnetic field compared with the perfectly aligned electrodes,Figure 3.

If the center of the coordinates is considered to be the center of the aligned electrodes, Ampere’s law can be summarized according to Equation (2):

(2) $$B\left(R\right)=\left\{\begin{array}{c} \frac{{µ}_{0}I}{2\pi {\left(r-\frac{\delta }{2}\right)}^{2}}R,\;R<r-\frac{\delta }{2}\\ \frac{{µ}_{0}I}{2\pi R},\;R>r-\frac{\delta }{2} \end{array}\right.$$

where *r* is the radius of the active face of the electrodes, usually 6 mm; *R* is the distance to the center of the generated magnetic field and *δ* is the magnitude of the misalignment in the direction of *R*.

To confirm the robustness and the influence of other mechanical defects, the behavior of the magnetic field generated for each of the different possible mechanical defects was studied [35], verifying that the influence of a misalignment was greater than any other mechanical defect studied.

In conclusion, what was sought in this last phase of the investigation was to apply the previous investigations in a real industry, presenting a system capable of being integrated into high-production lines of the automobile industry in an effective and efficient way.

## 3. Material and Methods

### Electrode Misalignment Detection Device

To measure misalignments, a device capable of measuring the magnetic field was developed. In addition, according to the objectives of this research, the device was also capable of being integrated within the idiosyncrasy of a factory, being easily installable and allowing interactions with the other components involved in production (e.g., PLC and databases).

**Figure 4:**
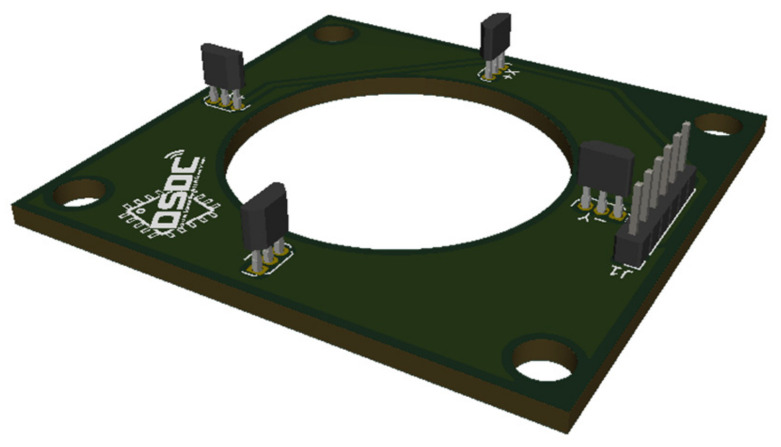
Hall effect sensor PCB.

The magnetic field measurements were carried out by means of four hall effect sensors, specifically SS49E [36] manufactured by the Honeywell Corporation. These were used in the device due to their range of measurements as well as their linearity and bipolarity. The measurements were made by the four hall effect sensors, which were located antiparallel in such a way that the values were collected in the four Cartesian axes, as shown on the PCB in Figure 4.

**Figure 5:**
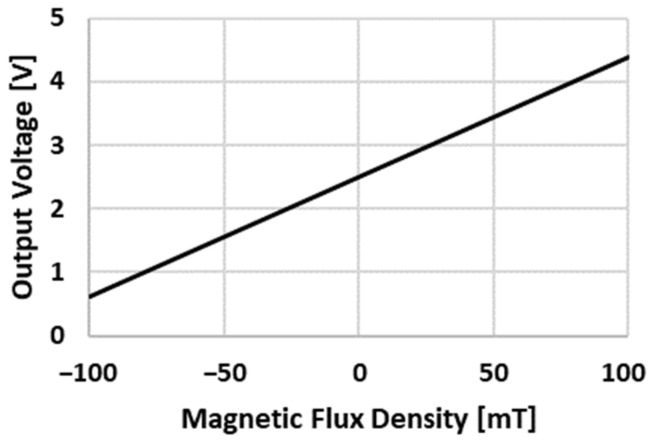
SS49E transfer characteristics.

To obtain the magnetic field value, the following protocol was followed. The electrodes were milled after a certain number of welding points in order to keep their geometry constant. Once the electrodes were milled, they were short-circuited in the middle of the circumference of the PCB. When the electrodes were in position, a current then flowed between them, generating a magnetic field that was measured by the four hall effect sensors. These four sensors measured the magnetic field and converted that value into a voltage, according to Figure 5. The objective of the measurement of the magnetic field was not to quantify it, but to determine the variations in the acquired values; therefore, the use of the unit of the magnetic field in mT or in mV was indifferent. For this reason, and throughout this paper, the value in volts of the magnetic field was taken as the unit of measurement of the magnetic field.

**Figure 6:**
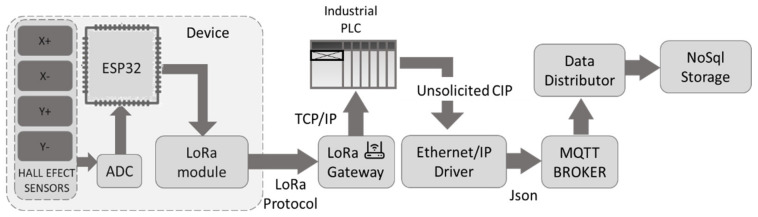
Scheme for sending the data collected during the check.

The magnetic field values were acquired by a microcontroller based on the ESP32 that received the information, calculated the magnetic field difference in each axis and then sent the values to the PLC. Figure 6 shows the communication protocol between the device and the database. The data were acquired by the device and sent to the gateway through the LoRaWAN communication protocol, which in turn sent the data to the PLC. Finally, the PLC acted as a gateway between the LoRa gateway and the database in which the data were stored to be analyzed.

## 4. Experiment and Results

For the method and device performance validation, the device was installed in a real production station so that it could be validated under real operating conditions. The data collected during the misalignment check were stored and analyzed in the database. Initially, a calibration of the data was carried out on the basis that the initial data corresponded with the correct operation of the welding gun. Filtering and outlier elimination methods were applied to these data.

As mentioned above, the magnitude of the magnetic field depended on the direction in which the misalignment occurred. Thus, to determinate the state of the misalignment, the magnetic field values collected on the *x*-axis and *y*-axis were independently analyzed.

**Figure 7:**
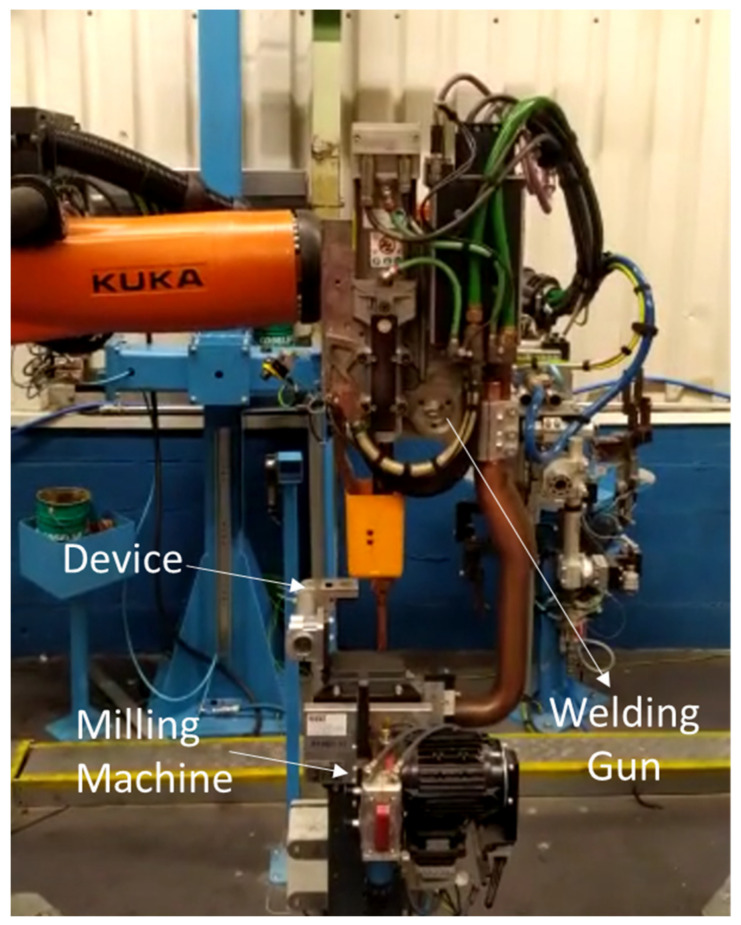
Location of the device in the production line.

To check for a correct operation, an ARO 3G gun with 16 mm type-F electrodes was used, according to DIN EN ISO 5821 [37]. This experiment was carried out inside a production welding gun to approximate the study to a completely real case, as can be seen in Figure 7.

**Figure 8:**
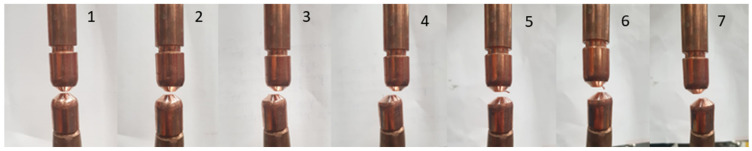
Alignment status for each of the experimental cases.

To verify a real operation, according to the theory and the simulations carried out in previous investigations [32], an experiment was carried out that modified the alignment of the electrodes. In total, seven different cases of alignment on the *x*-axis were performed, as shown in Table 1 and Figure 8.

**Figure 9:**
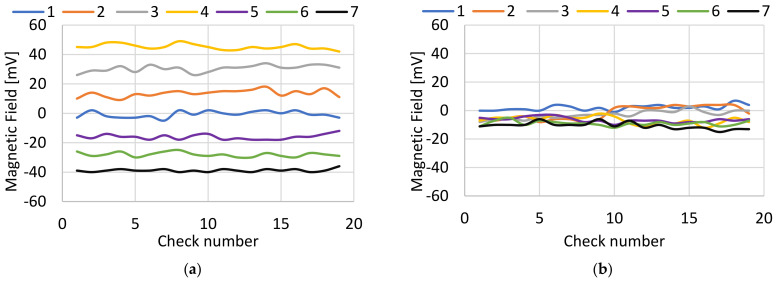
Experimental result: magnetic field generated for each of the cases. (**a**) Magnetic field measured on the *X*-axis. (**b**) Magnetic field measured on the *Y*-axis.

For each of the cases described above, a series of checks were made to verify that the variations in the magnetic field generated depended on the state of the alignment of the electrodes. As mentioned previously, the misalignment detection device calculated the average magnetic field of each of the sensors for each check and sent the difference between the axes to the welding database. In Figure 9, two different graphs can be seen, one corresponding with the values of the magnetic field on the *x*-axis (Figure 9a) and the other corresponding with the values of the magnetic field on the *y*-axis (Figure 9b).

Figure 9a shows how Case 1 corresponded with the magnetic field data for a few aligned electrodes, with values close to 0 mV due to the initial calibration. If Cases 2, 3 and 4 were compared, as the misalignment on the *x*-axis increased, the magnetic field values also increased. This was because each time the center of the magnetic field was closer to the measurement sensor located on the x+, it was increasingly further away from the measurement sensor located on the *x*-axis. When Cases 5, 6 and 7 were observed, the misalignment occurred in the reverse direction—that is, in the direction of the measurement sensor located at x-and the absolute value of the magnetic field increased. On the other hand, as shown in Figure 9b, it could be seen that the magnetic field values were very similar, regardless of the case of misalignment.

**Figure 10:**
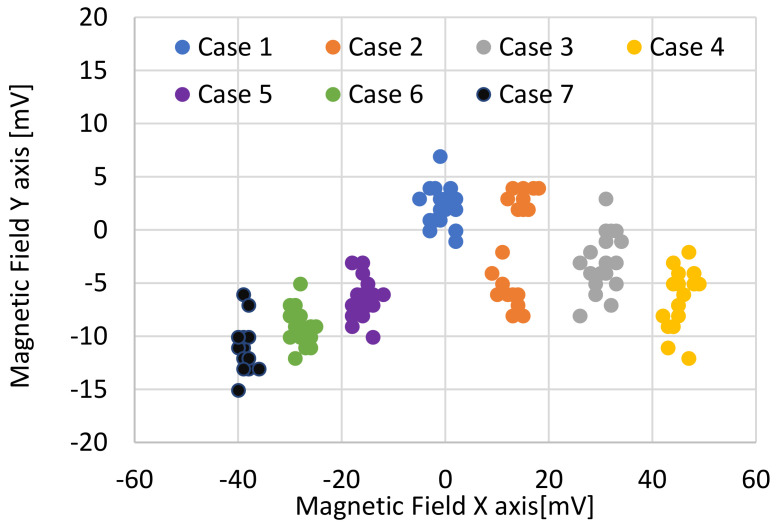
Magnitude of X-Y magnetic field generated by each case.

The global behavior of the generated magnetic field is reflected in Figure 10. This figure shows the values of the magnetic field measured on the *X*-axis and *Y*-axis. It could be seen that there was a variation mainly in the values of the magnetic field on the *X*-axis, varying from the maximum value of 50 mV to the minimum value of about −40 mV. From Figure 8 and Figure 9, it could be concluded that there was a direct relationship between the state of alignment of the electrodes and that this mainly affected the magnetic field measurements taken in the axis in which the misalignment occurred.

Several statistical variables such as the mean and the standard deviation could also be obtained from these calculated data. This statistical study allowed the analysis of the behavior of the data and their variability within the same alignment state. These statistical calculations are shown in Table 2.

Two main conclusions could be drawn from Table 2. First, the averages of the values on the x-axis and *y*-axis served to confirm, once again, that the method was able to detect changes in the alignment of the electrodes; and second, that the dispersion was similar, regardless of the case of the particular study. This guaranteed an acceptable repeatability of the device measurements, thus avoiding false alarms caused by dispersion in the measurements. Despite this, in Case 2, it could be seen that there was a greater variation in the data; this may have been due to variations in the milling characteristics [35].

## 5. A Real Case Implementation

Once a device capable of detecting the misalignment of electrodes by means of magnetic fields had been designed, and following the objectives sought with this research, a system for the detection of misalignments in high-production lines was designed.

Based on this objective, an acquisition, processing and sending alarm system was designed for the installation of devices in a production line.

**Figure 11:**
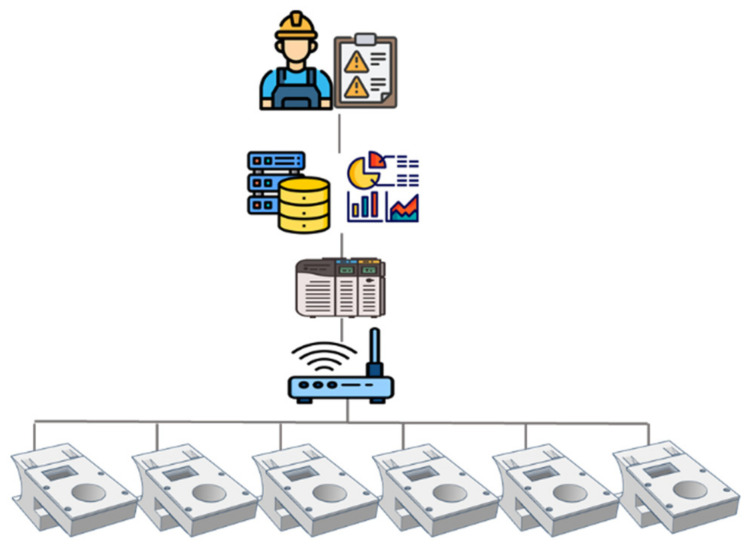
Alarm-sending scheme.

For this purpose, the system designed by Garcia et al. [38] for the communication of industrial equipment with an analysis database was followed. In this particular case, the data came from the misalignment detection sensors; these were sent through LoRa to the gateway that provided the data to the PLC. Once the data were in the PLC, they were sent to the database for storage and a subsequent analysis. This is summarized in Figure 11.

The alarm-sending system followed the following process. First, during the installation of the devices, the state of the alignment of the electrodes was visually checked in such a way that it could be established that the initial measurements corresponded with an electrode in good condition. These values served to eliminate the offset of the measurements in such a way that when the electrodes were aligned, the magnetic field values in *x* and *y* were close to zero.

As has been seen throughout the previous sections, the magnitude of the magnetic field depended on the direction in which the misalignment originated. For the validation of the method, it was necessary to independently analyze the *x* and *y* components of the magnetic field. However, for the misalignment detection and alarm-sending system, it was only necessary to know when the magnetic field varied regardless of the direction in which the misalignment occurred. This was because the operator would have to repair the condition of the electrodes regardless of the direction of the misalignment. Therefore, the magnitude of the magnetic field vector was calculated from the *x* and *y* values, which was the value on which the alarms and pre-alarms were set.

**Figure 12:**
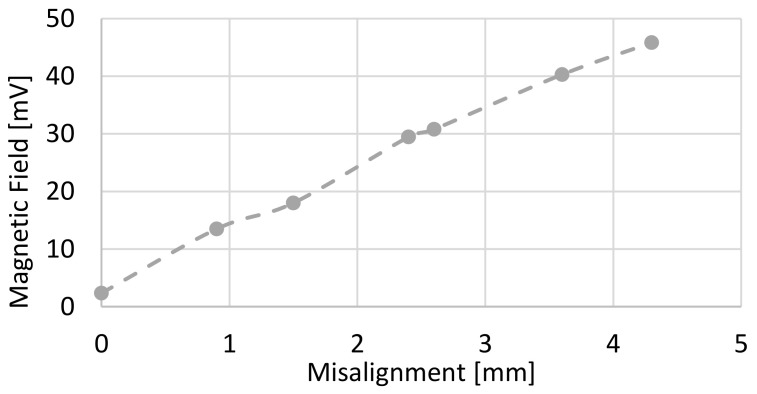
Magnitude of the magnetic field as a function of the misalignment of electrodes.

The method of setting the alarms was based on previous experimentation. Figure 12 was obtained from the calculation of the value of the vector for each of the cases. This figure represents the value of the magnitude of the magnetic field vector for each of the misalignments carried out in the Experiment and Results section.

Based on the values obtained in Figure 12, the alarm and pre-alarm values were established. Assuming that the acceptable misalignment limit was 1 mm, a pre-alarm value could be obtained from the equation of the straight line, giving an approximate result of 13.7 mV. Regarding the alarm, it was considered to be a serious status of misalignment above 2 mm; thus, the alarm value was set at 23.8 mV.

**Figure 13:**
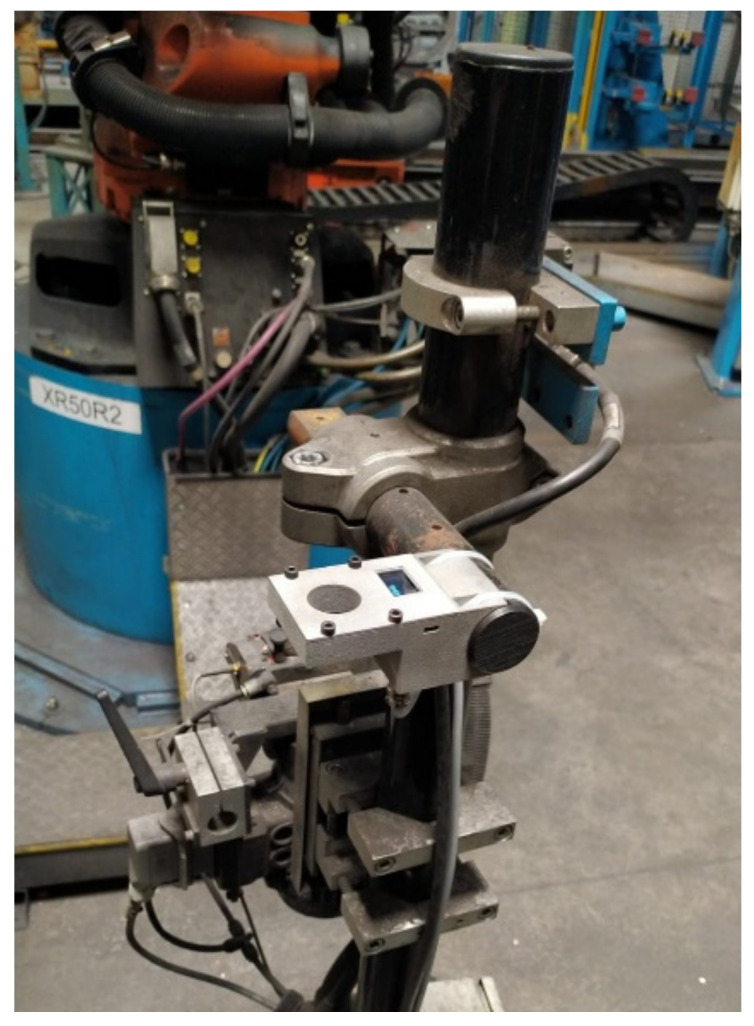
Production line device installation.

In this first implementation, seven devices were installed within a production line to finally validate the complete system for detecting changes in the alignment of the electrodes (Figure 13). For the start-up of the devices, the initial alignment of the seven welding guns was checked, creating the offset for the start of the alarm system.

The welding robot was programmed so that after carrying out the entire process of milling the electrodes, it performed an alignment status check after a certain cycle of points. To carry out the check, the same configuration as in the experimental test was followed, setting the check values at 8 kA for 200 ms.

**Figure 14:**
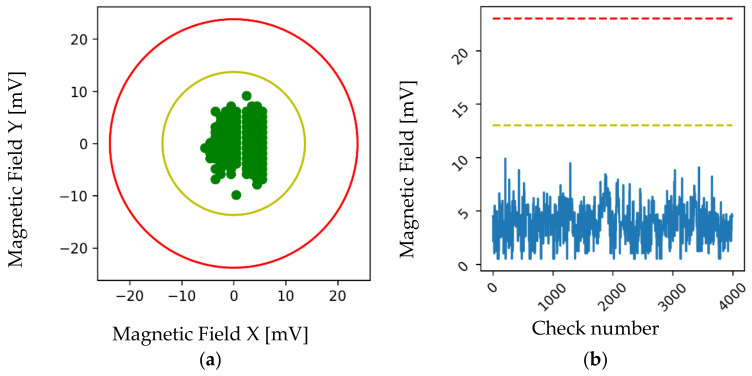
Data obtained from a welding gun in good condition from a production line. (**a**) Magnetic field axes *X* and *Y*. (**b**) Data series of the magnitude of the magnetic field.

For the analysis of the alarm system, two different graphs were obtained for each of the devices. Figure 14a shows the magnetic field values of *x* and *y* in the coordinate axis during a given period, also showing the pre-alarm threshold in yellow and the alarm threshold in red. Figure 14b shows the series of checks carried out in which temporary trends and behaviors can be observed. The alarm and pre-alarm limits established in the previous section are shown in the same way. As can be seen, during all the checks, the magnetic field values were within the thresholds of good operation in such a way that it could be stated that this welding gun did not suffer from electrode alignment problems.

**Figure 15:**
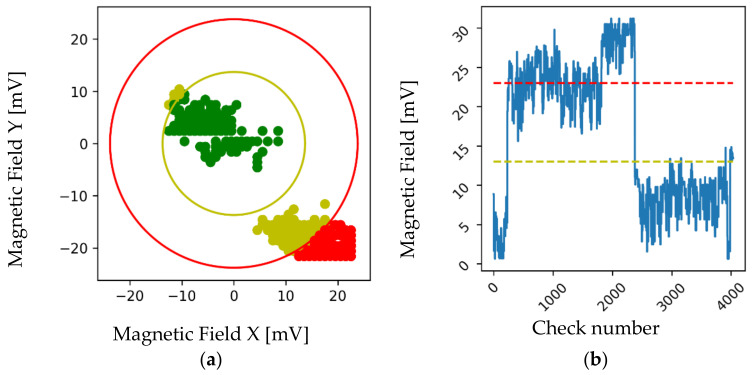
Data obtained from a misaligned welding gun from a production line. (**a**) Magnetic field axes *x* and *Y*. (**b**) Data series of the magnitude of the magnetic field.

In contrast to the graphs of Figure 14, the data obtained for a welding gun with alignment problems are shown in Figure 15. Figure 15a shows how many of the checks carried out obtained a value greater than the pre-alarm threshold, classified as abnormal behavior. Likewise, a number of points were located above the alarm threshold; these became a critical work area and, therefore, required corrective maintenance actions.

In the same way, observing Figure 15b, at around check 200 there was a significant increase in the values of the magnetic field, exceeding the pre-alarm threshold established at around 13.8 mV. This increase was due to a collision between the robot and the milling machine that ended in a mismatch in the alignment of the electrodes. As the checks continued, at around check 2000 the condition of the electrodes worsened and all the checks were above the alarm threshold. Once the maintenance tasks for the reorientation of the electrodes to their original alignment position had been carried out, at around the 2500 check the magnetic field values were positioned below the pre-alarm and alarm values, thus returning them to normal operating values.

In conclusion, when comparing the behavior of the welding guns in the production lines, it could be determined that this system met the requirements to be implemented in production lines, as it was able to differentiate between the behaviors of perfectly aligned guns, guns with the beginning of a misalignment and totally misaligned guns.

## 6. Conclusions

This paper investigated the relationship between the alignment of electrodes and the magnetic field generated by welding guns in order to find a reliable method for the detection of this mechanical defect in high-productivity production lines, specifically in the car industry. The main conclusions that were drawn on the basis of the study were:

(1) The contact area of the electrodes varied when there was a misalignment of the same; this generated a variation in the magnetic field generated when a current flowed through them.
(2) When measuring the magnetic field always at the same point, the value of the magnetic field changed depending on the amount of misalignment present between both electrodes of the welding gun.
(3) It was possible to measure the magnetic field of a welding gun with the proposed device, obtaining magnetic field values in the Cartesian axes.
(4) A reliable protocol for sending alarms was proposed, sending the values through LoRa to the data analysis software. Based on the experimentation, alarm thresholds were established that were capable of determining when a misalignment occurred.

In short, a real solution was given in this paper to the problem of misalignments in production lines, not only presenting a method capable of detecting misalignments in real-time, but also a system that can easily be integrated into high-production lines of the automobile industry, thus presenting real cases of success of the implantation.

## Figures and Tables

**Table 1:** Summary of experimental cases based on alignment status.

Case	1	2	3	4	5	6	7
δ (mm)	0	0.9	2.6	4.3	−1.5	−2.4	−3.6

**Table 2:** Standard deviation and average for each axis in each experimental case.

Case	1	2	3	4	5	6	7
$\bar{x}$ (mV)	−0.8	13.4	30.7	45.2	−15.7	−28.1	−38.8
$\bar{y}$ (mV)	1.7	−1.8	−2.7	−6.8	−6.5	−9.2	−10.8
σ_*x*_ (mV)	2.2	2.5	2.3	1.9	2.2	1.6	1.0
σ_*y*_ (mV)	2.2	4.7	2.9	2.7	1.8	1.8	2.4

## Data Availability

Not applicable.
